# Supplementary material for: Schistosoma japonicum leishmanolysin SjLLPi1 facilitates the invasion of cercariae into the host skin
Source: PLoS Pathog. 2025 Aug 26;21(8):e1013446. doi: 10.1371/journal.ppat.1013446 (PMC12410865; doi:10.1371/journal.ppat.1013446)
Supplement: S1 Data — (DOC) [file ppat.1013446.s001.doc]

**Supporting Information**

***Schistosoma japonicum* leishmanolysin SjLLPi1 facilitates the invasion of cercariae into the host skin**

**Short title: *Schistosoma japonicum* SjLLPi1 facilitates cercariae invasion**

Fanyan Chen^1,2†^, Bingkuan Zhu^3†^, Yu Fang^1†^, Zilüe Li^1†^, Zhigang Lei ^1†^, Zechao Xue^1^, Tao Shen^1^, Sha Zhou^1^, Xiaojun Chen^1^, Lei Xu^1^, Yalin Li^1^, Jifeng Zhu^1^*, Wei Hu^3^*, Chuan Su^1^*

**Materials and Methods**

**Treatment of BMDMs with different concentrations of SjLLPi1**

BMDMs derived from eight-week-old male C57BL/6 mice were exposed to recombinant SjLLPi1 at concentrations of 2.5, 5, 10, 20, or 40 μg/ml for 24 hours. PBS treated BMDMs were used as control. Total RNA was then extracted from the cells for the analysis of mRNA expression levels of TNF-α, iNOS, IL-6, IL-10, and Arg-1.

**Carmine staining of parasites**

Cercariae and schistosomula were fixed in AFA solution (2% acetic acid, 3% formaldehyde, 95% of 70% ethanol), rinsed with 70% ethanol, and stained overnight at 4 °C with 4% hydrochloric carmine (Leagene, Beijing, China). Excess stain was removed by immersion in acid alcohol (1 ml concentrated HCl in 99 ml 70% ethanol) for 20 s. Samples were then dehydrated in 80% and 95% ethanol (2 h each), 100% ethanol (1 h, twice), followed by xylene (10 min, twice). Samples were mounted with neutral balsam (Servicebio, Wuhan, China).

**Figure legends**

**S1 Fig. Macrophage activation induced by different concentrations of SjLLPi1.**

BMDM were isolated from mice and treated with different concentrations recombinant SjLLPi1, mRNA levels of (A) TNF-α, (B) iNOS, (C)IL-6, as well as (D) IL-10 and (E) Arg-1 were detected by real-time RT-PCR. Data were means ± SD of 3 samples from three independent experiments.

**S2 Fig. Morphology examination of schistosome larvae by carmine staining.**

(A) Cercariae were incubated with anti-SjLLPi1 antibody or mouse IgG or anti-HSP60 antibody for 10 min, morphology of cercariae was examined by carmine staining as described in the Materials and Methods (Scale bar, 50 µm). (B) Mice were infected with anti-SjLLPi1 antibody or mouse IgG or anti-HSP60 antibody treated cercariae, schistosomula were collected from the skin 30 min post-infection, morphology of schistosomula was examined by carmine staining as described in the Materials and Methods (Scale bar, 25 µm).
