# Supplementary figures and images for: Schistosoma japonicum leishmanolysin SjLLPi1 facilitates the invasion of cercariae into the host skin
Source: PLoS Pathog. 2025 Aug 26;21(8):e1013446. doi: 10.1371/journal.ppat.1013446 (PMC12410865; doi:10.1371/journal.ppat.1013446)

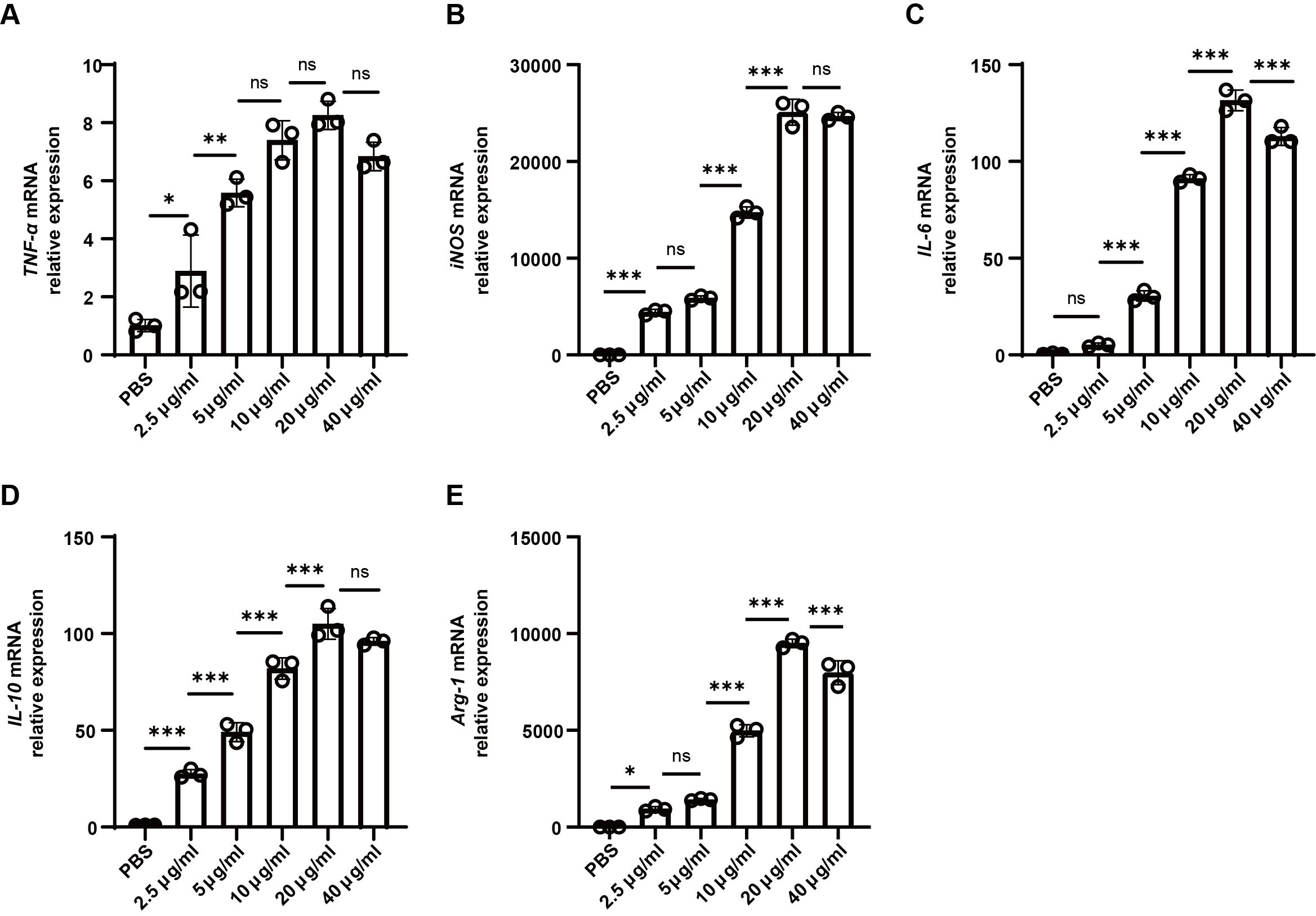

Supplement: S1 Fig — BMDM were isolated from mice and treated with different concentrations recombinant SjLLPi1, mRNA levels of (A) TNF-α, (B) iNOS, (C)IL-6, as well as (D) IL-10 and (E) Arg-1 were detected by real-time RT-PCR. Data were means ± SD of 3 samples from three independent experiments. (TIF) [file ppat.1013446.s003.tif]

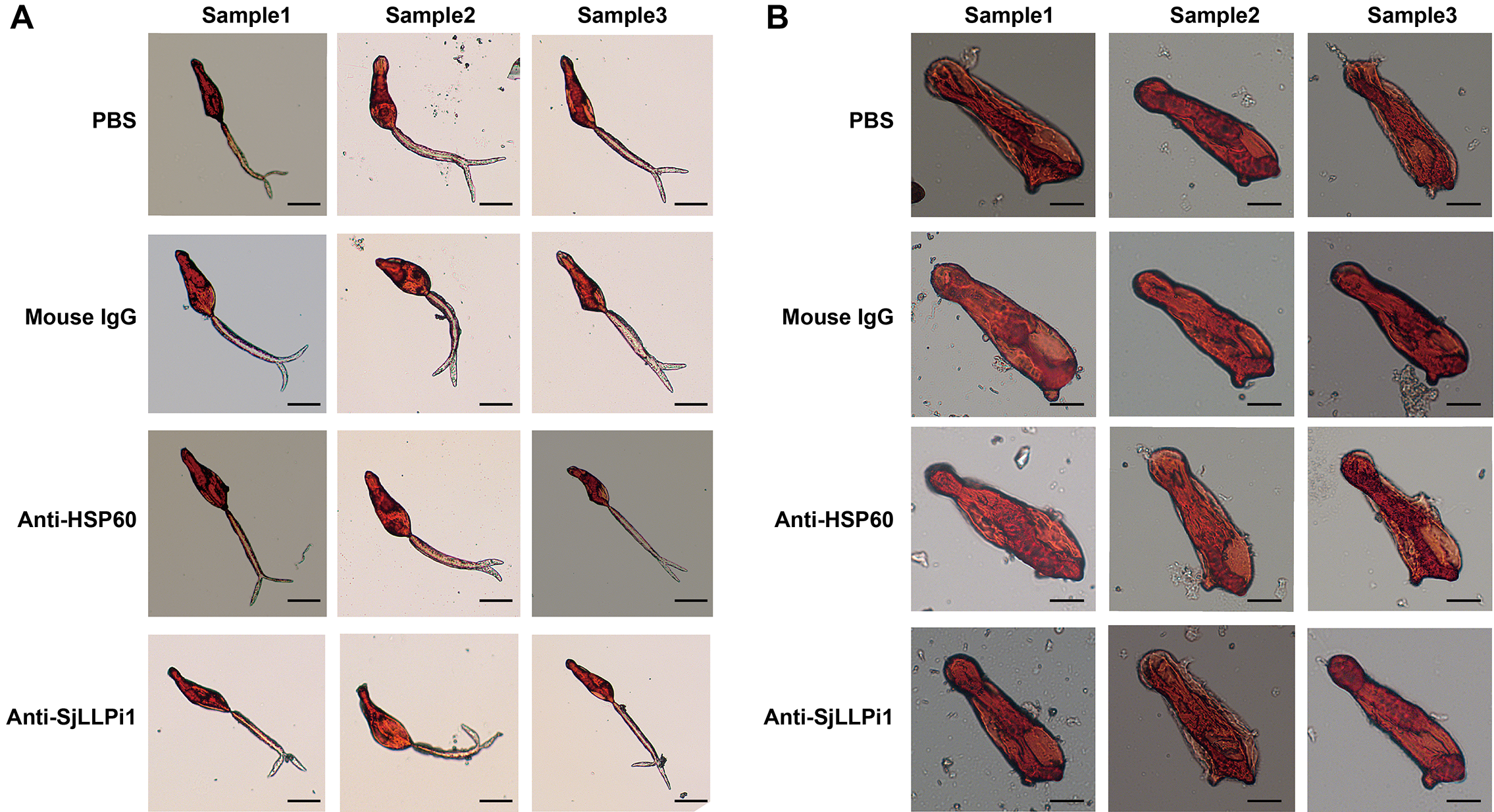

Supplement: S2 Fig — (A) Cercariae were incubated with anti-SjLLPi1 antibody or mouse IgG or anti-HSP60 antibody for 10 min, morphology of cercariae was examined by carmine staining as described in the Materials and Methods (Scale bar, 50 µm). (B) Mice were infected with anti-SjLLPi1 antibody or mouse IgG or anti-HSP60 antibody treated cercariae, schistosomula were collected from the skin 30 min post-infection, morphology of schistosomula was examined by carmine staining as described in the Materials and Methods (Scale bar, 25 µm). (TIF) [file ppat.1013446.s004.tif]
