# Supplementary material for: Schistosoma japonicum leishmanolysin SjLLPi1 facilitates the invasion of cercariae into the host skin
Source: PLoS Pathog. 2025 Aug 26;21(8):e1013446. doi: 10.1371/journal.ppat.1013446 (PMC12410865; doi:10.1371/journal.ppat.1013446)
Supplement: Attachment — Submitted filename: Response letter.docx [file ppat.1013446.s006.docx]

We appreciate the helpful and constructive comments of the reviewers. Our point-by-point responses to the reviewers’ comments are described below:

**Part I - Summary**

Reviewer #1:

The novelty, significance, planning and general execution are remarkable. The weakness are due to the fact that the important information generated may not be translated into a drug or vaccine for the dreadful disease.

Response: We thank the reviewer’s comments. Indeed, the SjLLPi1 investigated in our study has not yet been translated into a drug or vaccine, which may require a prolonged period of research and development, and we do have future plans.

Reviewer #2:

Chen et al. have generated an antibody against the SjLLPi1 and show that preincubation with the antibody targeting the leishmanolysin reduces the enzymatic activity of the protein and that the infection is reduced in a mouse model 42 and 7 days post infection. Moreover, they show that a lower number of schistosomula are identified in the skin 30 minutes post-infection, suggesting that the leishmanolysin may play a role in the penetration of the skin.

Response: None.

Reviewer #3:

This manuscript describes the initial characterization of Schistosoma japonicum leishmanolysin SjLLPi1. This is novel because while leishmanilysin enzymes have been described in S. mansoni, they have not been described in S. japonicum. These authors successfully identified SjLLPi1, expressed it as a recombinant protein, and generated monoclonal antibodies which were used to localize it to S. japonicum cercariae heads in the acetabular glands as well as demonstrate it's role in skin penetration in a mouse model. Some weaknesses include details that need to be clarified in the materials and methods. Overall, this is a well constructed manuscript describing a key enzyme in S. japonicum facilitating infection, and a potential therapeutic target.

Response: We thank the reviewer’s comments and helpful suggestions. We have improved our revised manuscript especially the Materials and Methods section.

**Part II – Major Issues: Key Experiments Required for Acceptance**

Reviewer #1: None.

Reviewer #2:

1. The authors have identified and expressed S. japonicum leishmanolysin SjLLPi1 and identified this as a protein expressed in cercariae only.

- Previous studies (Hambrook et al PPAT 2018) using S. mansoni indicate that the protein is expressed also early in miracidia, applying a role in the invasion of the snail. The authors do not refer to this paper, which should be considered. Also, a more detailed kinetics study could be considered to claim that the SjLLPi1 is only in the cercariae.

Response: We would like to thank the reviewer for the suggestions. Previous study on *S. mansoni* have shown that the leishmanolysin is also expressed, detected by both quantitative RT-PCR and western blotting, at the early miracidia stage [1], suggesting a role in the invasion of the intermediate snail host. However, in our study, SjLLPi1 mRNA was primarily detected at the sporocyst stage by RNA-seq and quantitative RT-PCR, whereas the SjLLPi1 protein could only be detected in the cercarial stage of *S. japonicum* by western blotting. This indicates that the leishmanolysin of *S. japonicum* may differ from that of *S. mansoni*, with its protein expression predominantly in the cercarial stage, potentially contributing to the invasion of the definitive mammal hosts. This issue has been addressed in the Discussion section of the revised manuscript at page 26 lines 23-25 and page 27 lines 1-7, and we cited this reference as suggested by the reviewer.

We performed western blotting to detect SjLLPi1 protein at egg, miracidium, sporocyst, and cercaria stages, but the protein was only detected at cercariae stage. Considering the primary aim of our study is to determine whether SjLLPi1 is produced in cercariae and functions as a protease for definitive host skin penetration, whether SjLLPi1 expression begins at the late sporocyst stage, early cercarial stage, or later during cercarial maturation is likely not only much bigger number of snails needs but also beyond the scope of or not so important in this study. Therefore, a more detailed kinetics western blot study was not conducted in this study.

[1] Hambrook JR, Kabore AL, Pila EA, Hanington PC. A metalloprotease produced by larval Schistosoma mansoni facilitates infection establishment and maintenance in the snail host by interfering with immune cell function. PLoS pathogens. 2018;14(10):e1007393.

2. A dose dependent activity of the SJLLPi should be shown in figure 2.

Response: As suggested by the reviewer, we have performed additional experiment, which showed a significant dose-dependent enzymatic activity of SjLLPi1. This result has been included in our new Figure 2E and described in the Results section of the revised manuscript at page 20 lines 8-10.

3. The enzymatic activity was tested in vitro using a recombinant enzyme. However, it is not clear whether the effect of the antibody is on the inhibition of the enzyme or if the coating of the cercariae with antibodies directed against the parasite per se increases the killing of the worm.

- Ideally, the expression of the protein would be inhibited using molecular methods to avoid coating the parasites with antibodies, which may effectively trigger the immune system. Previous data suggest that sporocysts can be targeted by siRNAs (Hambrook, 2018).

- Alternatively, can the enzymatic effect of the cercariae be tested to show the impact of antibody treatment on degradation, as shown in Figure 2D?

Response: As mentioned by the reviewer, certain genes expression in *S. mansoni* sporocysts had been successfully targeted by adding siRNA into medium during in vitro culture of sporocysts for 24 hours [1]. But in vitro cultured sporocysts cannot develop to cercariae for further study of definitive host skin penetration. We have thought about injection of siRNA into sporocysts within *Oncomelania hupensis*. However, applying this approach to *Oncomelania hupensis* is technically challenging due to the very small size of the snails, making injection difficult and harmful to the intermediate host. Therefore, we have decided to employ two approaches to analyze enzymatic effect of the cercariae, as well as the impact of antibody treatment on enzymatic effect of the cercariae: (1) We collected cercarial secretions and assessed their hydrolytic activity against the substrate, along with the inhibitory effect of anti-SjLLPi1 antibody. (2) We sonicated cercariae and collected the supernatant to evaluate its hydrolytic activity, and the inhibitory effect of anti-SjLLPi1 antibody.

Our results showed that both the cercarial secretions and the sonicated supernatant exhibited hydrolytic activity toward the substrate, and this activity was significantly inhibited by anti-SjLLPi1 antibody. These findings have been presented in Figure 4B and Figure 4C, and described in the Results section of the revised manuscript at page 21 lines 10-16.

[1] Hambrook JR, Kabore AL, Pila EA, Hanington PC. A metalloprotease produced by larval Schistosoma mansoni facilitates infection establishment and maintenance in the snail host by interfering with immune cell function. PLoS pathogens. 2018;14(10):e1007393.

4. As stated above, coating the parasite with antibodies could affect the immediate immune response in the skin. If the SjLLPi1 cannot be targeted by molecular methods, a control for the effect of specific antibodies would be appropriate. For example, could the antibody to SjLLPi1 be compared to another antibody that coats the cercariae but does not inhibit the enzyme? Alternatively, could the effect of IgG from an immunized mouse on cercariae be tested 30 minutes after infection? In both cases, it would be relevant to determine the effect of the coating and compare it with the effects of antibodies generated against SjLLPi1.

Response: We would like to thank the reviewer for comments and the suggestions. The reduction of worm in mice after using anti-SjLLPi1 antibody may also resulting from the “vaccine-like effect” of anti-SjLLPi1 antibody. In all experiments involving the use of the anti-SjLLPi1 antibody, we had included a mouse IgG isotype control antibody, and we also realized that it did not serve the same purpose as what the reviewer referred to as "IgG from an immunized mouse."

Heat shock protein 60 (HSP60) is a constitutively expressed protein throughout all developmental stages of schistosomes [1, 2, 3]. To serve as a control antibody that may coat the cercariae but does not inhibit the enzyme, anti-HSP60 monoclonal antibody has been used. Cercariae were incubated with anti-HSP60 monoclonal antibody and used to infect mice. At 30 minutes post-infection, the number of schistosomula in the skin was quantified. No significant difference was observed between the anti-HSP60 monoclonal antibody-treated group and the mouse IgG control group. This result has been included in our new Figure 7D and described in the Results section of the revised manuscript at page 23 lines 21-25 and page 24 lines 1-5.

[1] Tielens AG, Heuvel Jvd, Eden Wv. Schistosoma mansoni: an HSP60 homologue is constitutively expressed in cercariae, adults, and sporocysts. 1993.

[2] Zhou S, Jin X, Chen X, Zhu J, Xu Z, Wang X, et al. Heat shock protein 60 in eggs specifically induces Tregs and reduces liver immunopathology in mice with Schistosomiasis japonica. PLoS One. 2015;10(9):e0139133.

[3] Yang L-l, Lv Z-y, Hu S-m, He S-j, Li Z-y, Zhang S-m, et al. Schistosoma japonicum: proteomics analysis of differentially expressed proteins from ultraviolet-attenuated cercariae compared to normal cercariae. Parasitology research. 2009;105(1):237-48.

5. Importantly, the cercariae must penetrate the skin which likely would involve degradation of keratin. Can the effects of SjLLPi1 on keratin, be measured? If so, how would the SjLLPi1 compare to elastases previously described in S. japonicum? Such data would support the role of the enzyme in skin penetration. Alternatively, which layer of the skin would be targeted by SjLLPi1 during penetration?

Response: To investigate the proteolytic activity of SjLLPi1 against proteins from different layers of the skin, we have selected representative major structural proteins of skin layers as substrates to assess their susceptibility to hydrolysis by SjLLPi1. Specifically, keratins K1 and K10 were chosen to represent the epidermis, type IV collagen for the basement membrane, and type I collagen for the dermis [1, 2, 3]. The results demonstrated that SjLLPi1 showed clear hydrolytic activity against keratin K10 and type I collagen, but not against keratin K1 or type IV collagen. These findings suggest that the hydrolytic effect of SjLLPi1 on host skin may primarily target the epidermis and dermis. These results have been included in Figure 7E and described in the Results section of the revised manuscript at page 24 lines 6-14.

[1] Mikesh LM, Aramadhaka LR, Moskaluk C, Zigrino P, Mauch C, Fox JW. Proteomic anatomy of human skin. Journal of proteomics. 2013;84:190-200.

[2] Coulombe PA, Lee C-H. Defining keratin protein function in skin epithelia: epidermolysis bullosa simplex and its aftermath. Journal of investigative dermatology. 2012;132(3):763-75.

[3] Reilly DM, Lozano J. Skin collagen through the lifestages: importance for skin health and beauty. Plastic and Aesthetic Research. 2021;8:N/A-N/A.

Reviewer #3: No new experiments needed for this manuscript.

**Part III – Minor Issues: Editorial and Data Presentation Modifications**

Reviewer #1:

1- Immunofluorescence staining was not described in the Materials and Methods section and may not be replaced by the sentence" we immunofluorescently labeled the protein in schistosome cercariae". Please, use arrows to mark the anterior region and acetabular glands in Figure 3C.

Response: We apologize for the omission of the description of the immunofluorescence staining method and sincerely thank the reviewer for pointing it out. We have added a detailed description of this method in the Materials and Methods section in the revised manuscript at page 13 lines 19-25 and page 14 lines 1-7.

As suggested by the reviewer, we have added arrows in Figure 3C to mark the anterior region and acetabular glands, and provided corresponding annotations in the figure legend.

2- In the Materials and Methods, Collection of parasites section should be placed before Mice infection.

Response: We thank the reviewer for the suggestion, and we have placed Collection of parasites section before Mice infection.

3- Last section of the Results is misplaced and must be placed in the Results and Discussion sections before "antibody treatment reduces worm and egg burden in S. japonicum-infected mice".

Response: From the chronological perspective of the biological events, it is indeed reasonable to place the section on "cercarial penetration into host skin" before the section describing "antibody treatment reduces worm and egg burden in *S. japonicum*-infected mice," as cercarial invasion of the host skin occurs first, followed by parasite development into adult worms and egg production. We appreciate the reviewer’s suggestion in this regard.

However, after the first step of confirming the hydrolytic activity of SjLLPi1, the next step and also the most important step of our study was to determine whether SjLLPi1 facilitates an increase in adult worm burden within the definitive host. This step was essential to verify the biological relevance and significance of SjLLPi1 in schistosome infection. After that, the third step for us was to exclude the possibility that this effect was due to immune evasion. At last, we identified the promotion of cercarial entry via its hydrolytic activity as the underlying mechanism. This sequence of discovery reflects the order in which we elucidated the function of SjLLPi1 during host infection and polishes the novelty of our findings. Therefore, we think that it might be better for our story to keep the current order of the results presentation.

4- The authors are kindly requested to discuss, generalize, and/or explain this sharp discrepancy between mRNA production and protein expression in both the sporocyst and cercarial stages.

Response: We observed that *SjLLPi1* mRNA is highly expressed at the sporocyst stage, whereas the SjLLPi1 protein is predominantly expressed at the cercarial stage. And we have referred literature and provided an explanation for this discrepancy in the Discussion section (in the revised manuscript, page 26 lines 18-23): “Previous study [1] on *S. mansoni* used incorporation of the cytosine analog 5-ethynyl-uridine (EU) and fluorescence labeling to detect active transcription in the cercariae stage, the results showed that fluorescence signals were detected in less than 10% of the cercariae, indicated there is very little transcription in cercariae.” Therefore, many proteins required in cercarial stage may be transcribed at the earlier sporocyst stage.

[1] Roquis D, Lepesant JM, Picard MA, Freitag M, Parrinello H, Groth M, et al. The Epigenome of Schistosoma mansoni Provides Insight about How Cercariae Poise Transcription until Infection. PLoS Negl Trop Dis. 2015;9(8):e0003853. Epub 20150825. doi: 10.1371/journal.pntd.0003853. PubMed PMID: 26305466; PubMed Central PMCID: PMCPMC4549315.

5- Please discuss the limitations of the experiments using antibody-coated cercariae regarding antibody inhibitory activity and redundancy of proteases-mediated cercarial infection, based on the significant but limited effect of antibody coating of cercariae before skin invasion.

Response: We thank the reviewer for the suggestion. We agree that the observed reduction in host invasion following antibody-coating of cercariae, while statistically significant, was only partial, and this may reflect several limitations in our current experimental design. We have discussed this issue in the Discussion section of the revised manuscript (page 29 lines 1-8) as following: Although anti-SjLLPi1 antibody significantly reduced number of skin-stage larvae, it did not completely prevent cercarial invasion. This partial inhibition may result from incomplete neutralization of protease activity during dynamic process of protease secretion and host skin degradation. In addition, the secretion of multiple proteases by cercariae, such as serine, cysteine, and aspartic types [1], suggests functional redundancy, whereby inhibition of a single enzyme is insufficient due to compensatory mechanisms. Such redundancy likely represents an evolutionary strategy to ensure successful host entry under varying environmental or immune conditions.

[1] Liu M, Ju C, Du XF, Shen HM, Wang JP, Li J, et al. Proteomic Analysis on Cercariae and Schistosomula in Reference to Potential Proteases Involved in Host Invasion of Schistosoma japonicum Larvae. J Proteome Res. 2015;14(11):4623-34. Epub 20150929. doi: 10.1021/acs.jproteome.5b00465. PubMed PMID: 26370134.

6- Caution should be shown in the last sentence of the text as it is nearly impossible for the host to elicit memory immune responses (The privilege of vaccines) to the leishmanolysin at few to 48 hours after exposure to cercariae. Drugs also are not expected to be considered.

Response: We appreciate the reviewer’s comment. To avoid over-interpretation, we revised the sentence in the Discussion section of the revised manuscript (page 29 lines 16-18) as follows: This research provides insights into the role of SjLLPi1 in schistosome invasion and may inform future strategies aimed at interfering with early stages of host entry.

Reviewer #2:

In figure 6, the effects of the recombinant SjLLPi1 on macrophages are shown. Are the levels of SjLLPi1 used when stimulating macrophages biologically relevant levels? Please comment.

Response: Since SjLLPi1 is a protease secreted by the parasite into the local site of parasite–host interaction, it is difficult to accurately detect its local working concentration. We referred to multiple published studies in which proteins derived from schistosome cercariae were used to treat immune cells in vitro, and selected a concentration of 10 μg/mL, which was most commonly used [1, 2]. Prior to the formal experiments, we performed a concentration gradient analysis centered around 10 μg/mL, testing two higher and two lower concentrations. The results showed that SjLLPi1 promoted macrophage activation across all concentrations tested. Therefore, we selected 20 μg/mL, the concentration that elicited the most pronounced effect, as the working concentration in our experiments. These data have been included in Supporting Fig. 1 of the Supporting Information and are described in the Results section of the revised manuscript at page 23 lines 6-7.

[1] Yoshino TP, Brown M, Wu XJ, Jackson CJ, Ocadiz-Ruiz R, Chalmers IW, et al. Excreted/secreted Schistosoma mansoni venom allergen-like 9 (SmVAL9) modulates host extracellular matrix remodelling gene expression. Int J Parasitol. 2014;44(8):551-63. Epub 2014/05/27. doi: 10.1016/j.ijpara.2014.04.002. PubMed PMID: 24859313; PubMed Central PMCID: PMCPMC4079936.

[2] Sanin DE, Mountford AP. Sm16, a major component of Schistosoma mansoni cercarial excretory/secretory products, prevents macrophage classical activation and delays antigen processing. Parasit Vectors. 2015;8:1. Epub 2015/01/07. doi: 10.1186/s13071-014-0608-1. PubMed PMID: 25561160; PubMed Central PMCID: PMCPMC4297449.

What the representative morphology reflects should better indicated in figure 7 or the legend. Also, it would be informative to know if the antibody treatment affected the morphology or if that was similar in both groups.

Response: We thank the reviewer for the helpful comments. In Figure 7B and 7C, we have added corresponding explanations to the figure legend. To evaluate whether the antibody treatment affected cercarial morphology, we incubated cercariae with anti-SjLLPi1 antibody or control antibody for 10 minutes, then assessed the morphology of the cercariae by carmine staining. No obvious changes in morphology were observed following anti-SjLLPi1 antibody treatment. In addition, we infected mice with anti-SjLLPi1 antibody-treated cercariae and collected schistosomula from the skin 30 minutes post-infection. Carmine staining of these larvae revealed no apparent morphological differences compared to controls, suggesting that anti-SjLLPi1 antibody treatment did not affect schistosomula morphology during skin penetration. Results of carmine staining are shown in Supporting Fig. 2. These results are described in the Results section of the revised manuscript at page 23 lines 24-25.

It is more relevant to show the SD instead of SEM as the variation within the group is the concern. Preferably individual data (dots) rather than bars are shown.

Response: As suggested by the reviewer, we have added individual data points for each sample to all statistical graphs and replaced the presentation of SEM with SD.

Typos noted:

Leishmania major line 4, p 9 should be in italic.

Spelling line 15 p 15: Should be Phylogenetic.

Line 19 p 36 figure (7) text last C should be D.

Response: Thanks for the reminder. We have carefully reviewed the entire manuscript and corrected all typos.

Reviewer #3:

Several items should be addressed in the materials and methods to improve clarity of the manuscript, as listed below:

Materials and methods

1. Please briefly describe how S. japonicum cercaria were collected from Oncomelania hupensis infected with S. japonicum, and how the cercariae were prepared before infecting mice.

Response: Thanks for the reminder. *Oncomelania hupensis* were placed in dechlorinated water and exposed to light for 3 hours to stimulate the shedding of cercariae. The emerging cercariae accumulated at the water surface and were collected using a capillary pipette, then transferred onto a coverslip for subsequent mouse infection. For antibody treatment, anti-SjLLPi1 antibody or control antibodies was added to the cercaria-containing water at a final concentration of 0.5 mg/ml. The mixture was incubated for 10 minutes at room temperature prior to infection. We have added these descriptions in the Materials and Methods section of the revised manuscript at page 14 lines11-17.

2. Please describe the number of mice used per group for infection studies.

Response: We have added individual data points for each sample to all statistical graphs in the revised manuscript, which clearly displays the number of mice used per group in the infection studies. In addition, the number of mice per group has been specified in the figure legends.

3. Please briefly describe the percutaneous mouse infection procedure with S. japonicum, or provide a citation that gives more details of the infection procedure. Were mice anesthetized during the infection procedure? If so, what anesthetic was used? Was the skin depilated and cleaned prior to percutaneous infection?

Response: As suggested by the reviewer, we have added a description of the *S. japonicum* infection procedure in mice to the Materials and Methods section of the revised manuscript at page 14 lines 17-23. Briefly, mice were lightly anesthetized with isoflurane (2% for induction, 1% for maintenance). Abdominal fur was removed using an electric pet shaver to expose the skin, which was then moistened with dechlorinated water. Cercariae were collected using a capillary pipette and transferred onto a coverslip. Under a  stereomicroscope, the desired number of cercariae was counted. The coverslip containing the cercariae was then applied to the abdominal skin of the mouse. After 20 minutes, the coverslip was removed, completing the infection procedure.

4. For infection of mice with the SjLLPi1 antibody treated S. japonicum cercariae, how much SjLLPi1 or control mouse IgG antibody was used to pre-treat the cercariae? Were the cercariae washed after treatment with antibody to remove unbound antibody before infecting mice?

Response: We apologize for omitting the antibody concentration used for treating the cercariae. The working concentration of both the anti-SjLLPi1 antibody and the control IgG used to treat the cercariae was 0.5 mg/ml. We have added this information to the Materials and Methods section of the revised manuscript (page 14, line 16).

The cercariae were not washed prior to infecting the mice to keep any anti-SjLLPi1 antibody in working, considering the SjLLPi1 is a gland secreted protease in response to host skin stimuli [1].

[1] Ingram J, Knudsen G, Lim K, Hansell E, Sakanari J, McKerrow J. Proteomic analysis of human skin treated with larval schistosome peptidases reveals distinct invasion strategies among species of blood flukes. PLoS neglected tropical diseases. 2011;5(9):e1337.

5. For quantification of liver and intestinal eggs, please include a statement that mice were humanely euthanized, then tissues were collected post mortem. Also, at what time point after infection were tissues collected for egg quantification?

Response: Thanks for the reminder. We have added the following statement to the Materials and Methods section of the revised manuscript (page 15, lines 4-6): Mice were sacrificed by cervical dislocation under deep anesthesia with 5% isoflurane, and the whole liver and small intestine were collected post mortem.

The time point for egg quantification was 42 days post-infection, and this information has also been included in the Materials and Methods section of the revised manuscript (page 15, line 4).

6. Please describe the methods used for counting eggs.

Response: As suggested by the reviewer, we have revised the description of the egg quantification procedure in the revised manuscript (page 15, lines 4-19) as follows:

The whole liver and small intestine were isolated from *S. japonicum* infected mice post mortem, washed with pre-chilled PBS, dried, and weighed (denoted as W). The liver and small intestine were then cut into fragments and digested at 37°C in 20 ml of 5% KOH for 2 hours. The suspension was centrifuged at 1700 × g for 5 min, the pellet was washed, re-suspended in 1ml PBS for egg counting. Fecal samples from S. japonicum infected mice at 6 weeks post-infection were collected, weighed (denoted as W), dissolved in PBS, filtered through an 80-mesh filter, centrifuged at 1700 × g for 5 min, and the pellet were re-suspended in 1ml PBS for egg counting.

A 10-μl aliquot of the suspension was placed on a microscope slide, and all eggs within the 10 μl suspension were counted under a microscope (denoted as n). The number (denoted as N) of eggs per gram of liver, intestine, or feces was calculated using the following formula: N = n × 100 / W.

7. For the schistosomula collection, what buffer was used to perfuse mice to collect schistosomula?

Response: We performed perfusion of infected mice using PBS, and this information has been added to the Materials and Methods section of the revised manuscript (page 11, lines 15 and 19).

8. For generation of BMDM, how were red blood cells removed?

Response: We used a self-prepared red blood cell (RBC) lysis buffer to remove RBCs. We have added a description of the RBC removal procedure to the revised manuscript (page 15, lines 22-24) as follows: Cells were incubated with RBC lysis buffer (10 mM KHCO₃, 155.2 mM NH₄Cl, 99.4 μM EDTA-2Na) at 4°C for 3 minutes to remove RBCs.

9. How many replicates of BMDM were used for treatment with SjLLpi1 or controls?

Response: In the experiments involving treatment of BMDMs with SjLLPi1 or control, one sample per group was included in each independent experiment, and a total of three independent experiments were conducted. Thus, the final analysis was based on three samples derived from three independent experiments.

10. Please include a description of the immunophenotyping procedure on BMDM in the materials and methods section.

Response: We have added the immunophenotyping procedure on BMDMs to the revised manuscript (page 16, lines 4-9) as follows: BMDMs were resuspended in PBS containing 1% FBS and incubated with FITC-conjugated anti-mouse F4/80 antibody and APC-conjugated anti-mouse CD11b antibody at 4°C for 30 minutes. After staining, cells were analyzed by flow cytometry to determine the proportion of F4/80⁺CD11b⁺ BMDMs, which was used to assess the purity of the isolated macrophage population.

Results
1. In page 19, line 15, please correct the spelling of “Phylogenetic”.

Response: We thank the reviewer for pointing out the typo. The term “Hhylogenetic” has been corrected to “Phylogenetic” in the revised manuscript.
